# Manic Episode Following Dengue Fever: A Case Report

**DOI:** 10.7759/cureus.68630

**Published:** 2024-09-04

**Authors:** Syed Ali Bokhari, Noura Alabrach, Alma Al Mansour, Mostafa Abulmagd

**Affiliations:** 1 Psychiatry, Al Amal Psychiatric Hospital, Emirates Health Services, Dubai, ARE; 2 Emergency Medicine, Al Qassimi Hospital, Emirates Health Services, Sharjah, ARE

**Keywords:** infectious and parasitic diseases, neuropsychiatry, neurology, psychiatry, post-dengue, mania, psychosis, dengue fever (df)

## Abstract

Dengue fever, a mosquito-borne viral disease, can present with a variety of symptoms, ranging from mild flu-like illness to more severe conditions such as dengue hemorrhagic fever and dengue shock syndrome. Although neurological symptoms such as headaches, dizziness, and altered sensorium are more frequently observed, psychiatric symptoms such as euphoria, delusions, hallucinations, and aggression, though rare, can occur. We present the case of a previously healthy 22-year-old male from South Asia who developed manic and psychotic symptoms, including insomnia, irritability, grandiosity, and auditory hallucinations, following his recovery from dengue fever. His psychiatric symptoms emerged shortly after discharge and necessitated psychiatric intervention with olanzapine, a second-generation antipsychotic, chosen for its suitability in managing manic symptoms. This case underscores the importance of considering psychiatric evaluations in the management of dengue fever, especially in endemic areas. The pathophysiology of dengue’s neuropsychiatric effects remains complex and multifactorial, necessitating further research. This case report aims to highlight the potential for significant psychiatric manifestations post-dengue fever, advocate for increased clinical awareness and research to investigate any potential correlation between dengue fever and psychiatric symptoms, and improve patient outcomes.

## Introduction

Dengue fever, first identified by Benjamin Rush in 1789 and labelled "breakbone fever" due to its severe joint pain, is a mosquito-borne viral disease transmitted by the female Aedes mosquitoes, mainly *Aedes aegypti *and *Aedes albopictus*. Belonging to the Flaviviridae family, the dengue virus has four serotypes (DEN-1, DEN-2, DEN-3, DEN-4), with an incubation period of 3 to 14 days [1]. It can cause a range of symptoms from mild flu-like illness to severe conditions such as dengue hemorrhagic fever, dengue shock syndrome, and potentially death [2].

Neurological manifestations are common in dengue and can include headaches, dizziness, altered sensorium, confusion, lethargy, and coma. Less frequently, seizures, neck stiffness, Reye’s syndrome, meningoencephalitis and paresis may occur. These central nervous system (CNS) symptoms can appear either before or after the emergence of hemorrhagic symptoms, primarily due to plasma leakage into serous spaces (third space fluid loss) and abnormal hemostasis, leading to hypovolemic shock and hemorrhage in various organs. Acute liver failure is also a significant factor contributing to CNS manifestations due to hepatic encephalopathy [2].

Psychiatric symptoms associated with dengue fever, although rare, can include excessive talkativeness, euphoria, increased activity, grandiosity, decreased need for sleep and food, irritability, restlessness, and outbursts of aggression [3,4]. Post-infectious sequelae such as hallucinations, delusions, amnesia, and dementia have also been reported [2,5,6].

The incidence of neurological and psychiatric symptoms in dengue fever in adults is difficult to estimate because such manifestations have only been described in selected compilations of case series or case reports [5,7-9].

It is critical for healthcare providers, in patients presenting with a recent travel history to endemic areas and acute psychiatric symptoms, to consider dengue in the differential diagnosis [3].

We report this case to underscore the rare occurrence of post-dengue psychiatric symptoms and their management, which is scarcely documented in the literature. This study aims to highlight the need for further research, including longitudinal studies with representative samples, to explore the potential causal relationship between dengue fever and psychiatric manifestations. Understanding this connection is essential for developing appropriate management strategies and improving patient outcomes in clinical practice.

## Case presentation

A 22-year-old single male, from South Asia, employed in his family business, previously healthy, presented to the Emergency Department of a general medical facility in the United Arab Emirates (UAE) escorted by his family with a five-day history of fever, nausea, non-bilious vomiting, diffuse abdominal pain, headache, body aches, and hemoptysis for the past four days. He denied any epistaxis, diarrhea, or melena. The patient occasionally smokes cigarettes and consumes alcohol. However, he last drank alcohol three months prior to his presentation to the Emergency Department. He denied any recent travel history. Both the patient and his family confirmed that he does not have any past psychiatric history, nor is there any family history of psychiatric disorders. He is not suffering from any chronic medical illnesses and is not on any regular medications.

His initial vitals at triage were as follows: heart rate of 103 beats per minute, blood pressure of 114/70 mmHg, respiratory rate of 18 breaths per minute, oxygen saturation of 99% on room air, temperature of 38.8°C, and weight of 50 kilograms. On physical examination, the patient's Glasgow Coma Scale was 15/15. He did not have any pallor, jaundice, or lymphadenopathy but had mild signs of dehydration. His chest was clear with equal bilateral air entry and normal S1 and S2 heart sounds. His abdomen was soft with minimal diffuse tenderness, no organomegaly was noted, and no lower limb edema was present. Neurological examination was unremarkable with no focal neurological deficits. No petechial rash was noted.

Blood laboratory investigations showed a positive dengue virus IgM result. He had a white blood count of 4.99 x10^3^/mcL and thrombocytopenia with a platelet count of 40.00 x10^3^/mcL. He was thereafter transferred to a field hospital (specifically constructed for dengue fever cases) to be managed as a case of dengue fever. The patient received symptomatic treatment including intravenous (IV) fluids and acetaminophen 1 gram once daily, pantoprazole 40 mg once daily, and ondansetron 4 mg pro re nata (PRN) (Table 1). He was discharged after six days, afebrile, with a platelet count of 104 x10^3^/mcL.

**Table 1:** Chronological list of medications administered to the patient during his hospitalization at both the dengue field hospital and the tertiary psychiatric facility. OD, once daily; HS, at bedtime; BID, twice daily

Medication	Drug Class	Route	Dosage	Frequency	Duration	Notes
Ondansetron	5-HT3 antagonist (for nausea and vomiting)	Intravenous	4 mg	OD	6 days	Administered during hospitalization at the field hospital.
Pantoprazole	Proton-pump inhibitor	Intravenous	40 mg	OD		
Acetaminophen	Analgesic and antipyretic	Intravenous	1,000 mg	OD		
Olanzapine	Second-generation antipsychotic	Oral	Initially 5 mg daily at bedtime, then titrated to 10 mg daily at bedtime	HS	10 days	Administered during hospitalization at the tertiary psychiatric facility.
Bromazepam	Benzodiazepine	Oral	1.5 mg	BID		

The patient presented again to the Emergency Department of the same general medical hospital within a week after discharge from the dengue fever field hospital, however this time with the chief complaint of a marked change in his behavior, which was significantly different from his usual self. His escorts reported that the patient, a day after his discharge (that is, around seven days prior to this presentation), began suffering from severe insomnia and an irritable mood with verbal aggression toward his family and roommates. These symptoms quickly escalated, manifesting as profound social disinhibition, decreased appetite, talkativeness, pressured and increased rate of speech, inflated self-esteem, and increased cigarette smoking. The patient also demonstrated impulsive behavior, such as excessive spending beyond his usual pattern. His Mental Status Examination (MSE) on presentation was significant for highly intense euphoric affect, incongruent with reported low mood, and grandiose attitude as well as ideations. He exhibited poor judgment and insight, as demonstrated by his expression of homicidal ideations, specifically mentioning the intention to use a knife against an unspecified individual.

These symptoms were particularly concerning due to the patient's absence of prior psychiatric history and their rapid onset following recovery from dengue fever. As a result, a non-contrast CT scan of the brain was conducted in the Emergency Department to rule out potential acute brain conditions such as encephalitis, encephalopathy, or stroke, especially in the context of dengue fever; the results were unremarkable for any intracranial pathology.

He denied physical complaints. His general examination and basic investigations (complete blood count, liver function tests, renal function tests, C-reactive protein, urea, and electrolytes) came back within normal limits. He was then transferred to a tertiary psychiatric facility, where he was admitted to an acute general psychiatry inpatient unit due to uncontrolled symptoms as well as high risk to himself and others. He required rapid tranquilization with intramuscular (IM) haloperidol 5 mg and IV diazepam 10 mg to sedate him prior to transfer.

To highlight suitability for his manic symptoms, he was started on olanzapine, a second-generation antipsychotic, at a dose of 5 mg once daily at bedtime (Table 1). His Young Mania Rating Scale (YMRS) at admission was 54. His total score on the Positive and Negative Syndrome Scale (PANSS) was 95 (PANSS Positive Subscale score: 37; PANSS Negative Subscale score: 12; PANSS General Psychopathology Subscale score: 46). He was diagnosed with bipolar and related disorder due to another medical condition, as per the Diagnostic and Statistical Manual of Mental Disorders, Fifth Edition, Text Revision (DSM-5-TR) [10].

While in the inpatient unit, he reported commanding auditory hallucinations. He also expressed persecutory delusions, fearing that he was being watched, he was being plotted against, and his safety was at risk. In addition, he exhibited grandiose delusions, claiming possession of immense wealth and the ability to purchase significant assets, such as entire buildings. He was observed to be overfamiliar with hospital staff and other co-patients. He was observed to be restless, talkative, and euphoric. Consequently, olanzapine was increased to 10 mg at bedtime and bromazepam to 1.5 mg twice a day (Table 1).

After 10 days of the inpatient stay, his symptoms completely subsided. His sleep pattern normalized, his appetite returned, both his manic and psychotic symptoms resolved, and he was no longer having any homicidal thoughts. He was subsequently discharged to his family on olanzapine 10 mg once daily at bedtime. His Young Mania Rating Scale (YMRS) at discharge was 2. During a follow-up appointment within one week of discharge, he was reported to be doing well, with no evidence of manic or psychotic symptoms. He was eating and sleeping well with no abnormal behavior noted. He was back at work and had returned to normal baseline functioning.

## Discussion

The pathophysiology underlying neuropsychiatric manifestations in dengue fever is complex and multifactorial. Prolonged shock, metabolic acidosis, and severe disseminated intravascular coagulopathy often lead to hepatic and brain dysfunction, contributing to the neurological and psychiatric symptoms observed in dengue patients [2,11]. Although neurological manifestations such as encephalitis and encephalopathy are well-documented, the occurrence of psychiatric symptoms such as mania and psychosis remains exceptionally rare [12,13].

A case report from India reported the development of manic symptoms in a patient following dengue fever, despite the absence of any prior psychiatric history. This suggests an organic basis for the mania, possibly linked to cerebral hypoxia as a result of transient conditions during the infection [11]. Such cases emphasize the need for clinicians to be vigilant about the potential for psychiatric symptoms following dengue fever, even in patients without a history of psychiatric disorders.

The mechanisms by which dengue fever leads to psychiatric symptoms are not entirely understood. Several theories propose that viral encephalitis, metabolic disturbances, and neuroinflammatory processes might play significant roles. For instance, the immune response to the dengue virus, characterized by the release of cytokines and other inflammatory mediators, can disrupt normal brain function, leading to psychiatric manifestations [3,6].

A systematic review of the literature found that while anxiety and depression are commonly observed during the acute phase of dengue infection, most of these symptoms tend to diminish during the recovery phase. However, there are also occasional reports of post-dengue cases presenting with mania, acute polymorphic psychosis, prolonged depression, and catatonia, several months post-infection. One such case involved a patient who developed significant overactivity, excessive talkativeness, religious preoccupations, and auditory hallucinations following complete remission from dengue fever. These symptoms, which developed without any prior psychiatric history, suggest that the dengue virus may induce psychiatric conditions through neuroinflammatory mechanisms [1]. This delayed emergence of psychiatric symptoms underscores the necessity for continuous psychiatric assessment and support for dengue patients [5,6].

The necessity of identifying and addressing psychiatric symptoms in dengue patients is paramount. With the global rise in dengue fever cases, there is an urgent need for heightened clinical awareness. This includes the United Arab Emirates (UAE), where concerns over dengue fever emerged after the heaviest rain on record caused flooding in several UAE neighborhoods, creating ponds of stagnant water that potentially became mosquito breeding sites. This led to a huge rise in dengue cases in recent months. The Ministry of Health and Prevention (MOHAP) played a huge role in mapping and wiping out mosquito breeding sites across the country using the latest GPS technologies. Such timely identification and treatment of dengue fever and its post-infection psychiatric sequelae are vital for enhancing patient outcomes. This is especially critical in dengue-endemic regions around the world, as these areas face the highest disease burden and may have limited healthcare resources [3].

To bridge the knowledge gap surrounding the neuropsychiatric complications of dengue fever, further research is required to uncover the prevalence, underlying mechanisms, and specific treatment strategies for these cases. Investigations should prioritize exploring the roles of neuroinflammatory processes, immune responses, and other contributing factors to enhance our understanding of the mechanisms through which dengue fever induces psychiatric symptoms. Additionally, longitudinal studies are crucial for tracking the long-term psychiatric outcomes associated with dengue infection and identifying effective interventions to manage these neuropsychiatric symptoms, thereby significantly improving patient care and clinical outcomes [6].

## Conclusions

This case report highlights a rare instance of psychiatric symptoms, specifically a manic episode, in a previously healthy young male following dengue fever. The patient developed rapid onset of symptoms, including insomnia, irritability, grandiosity, paranoia, and hallucinations, shortly after recovering from dengue, necessitating immediate psychiatric intervention with an antipsychotic medication, which successfully resolved the symptoms. The pathophysiology underlying these psychiatric manifestations remains complex and likely involves neuroinflammatory processes or immune responses. These mechanisms, while not fully understood, suggest that dengue fever can induce psychiatric conditions, even in individuals without a prior history of mental illness. The case also emphasizes the necessity for clinicians to remain vigilant for psychiatric symptoms in patients recovering from dengue fever, particularly in endemic regions where the incidence of the disease is high. Given the rising global incidence of dengue, integrating psychiatric assessments into post-dengue management could aid in early detection and treatment of neuropsychiatric complications. Our study highlights the need for further research, particularly in the form of longitudinal studies with representative samples, to establish causality to inform the development of appropriate management strategies for such patients, ultimately advancing patient care and improving patient outcomes.
